# Ambulatory blood pressure monitoring in children suffering from orthostatic hypertension

**DOI:** 10.1186/s12938-018-0530-4

**Published:** 2018-09-25

**Authors:** Yang Zhixiang, Wang Cheng, Xiang Jibing, Ge Bisheng, Xu Ming, Liu Deyu

**Affiliations:** 1 Department of Pediatrics, Lixian People’s Hospital in Hunan, Lixian, 415500 China; 2 Department of Pediatric Cardiovasology, Children’s Medical Center, The Second Xiangya Hospital of Central South University, Institute of Pediatrics of Central South University, Changsha, 410011 China

**Keywords:** Semiautomatic ambulatory blood pressure monitoring, Children, Orthostatic hypertension

## Abstract

**Background:**

It is particularly important to utilize appropriate blood pressure measurement methods to evaluate the changes of orthostatic hypertension (OHT) for children, and this study was designed to analyze the blood pressure type in OHT children with 24 h semiautomatic ambulatory blood pressure monitoring.

**Methods:**

Children who were diagnosed by head-up tilt table test as OHT patients (OHT group) and treated or hospitalized in the syncope specialist outpatient unit of the Second Xiangya Hospital of Central South University mainly for syncope or pre-syncope with unknown causes during the October, 2009 to September, 2013 were recruited in the study. Healthy children that came to the hospital for physical examination at the same time period according to age and sex were matched as control group. Semiautomatic ambulatory blood pressure monitoring of every child was recorded. The differences of daytime systolic (diastolic) pressure and night systolic (diastolic) pressure were calculated, and the average systolic pressure and diastolic pressure of the entire day, daytime and night were also calculated, respectively.

**Results:**

There were 23 boys and 17 girls in OHT group, aging (11.5 ± 1.9) years. There were 22 boys and 18 girls in the control group, aged (10.6 ± 2.4) years. The difference of daytime systolic pressure and night systolic pressure of the control group was higher than that of OHT group, while the average systolic pressure of the whole day, the average diastolic pressure of the whole day, the daytime average systolic pressure, the daytime average diastolic pressure, the night average systolic pressure and the night average diastolic pressure were higher than that of the control group (P > 0.05). The difference of daytime diastolic pressure and night diastolic pressure of the control group was higher than that of OHT group (P > 0.05). Most children of the OTH group had non-dipper blood pressure type (72.5%), while most children of the control group had a dipper blood pressure type (55.0%). In addition, the time domain SDNN and SDANN in the OHT group were higher than those in the control group (P < 0.01). And, the indicators including TP, ULF, VLF, and LF/HF were higher in the OHT group, when compared with control group (P < 0.01). Besides, in terms of subgroup analysis within the OHT group, the age difference between boys and girls was not statistically significant (P > 0.05). When compared with grils, the time domain SDNN increased (P = 0.003), and the frequency index TP, ULF, and VLF increased in boy group (P < 0.05).

**Conclusion:**

OHT Children’s autonomic nervous system showed dysfunction, and differences of systolic blood pressure between day and night were much lower than those of healthy children, and the main blood type was non-dipper blood pressure with circadian rhythm disappearing.

## Background

To our best knowledge, orthostatic hypertension (OHT) refers to normal blood pressure (BP) in the supine position, and suddenly raised blood pressure when standing or sitting [1]. As known to all, the normal BP is defined as systolic BP (SBP) less than 140 mmHg and diastolic BP (DBP) more than 90 mmHg at three consecutive consultations. And, OHT is defined as a drop of SBP less than 20 mmHg and/or DBP less than 10 mmHg at orthostasis [2]. It is noted that the BP should be assessed after 1 and 2 min of standing from a supine position. With respect to the physiological mechanism of OHT, it is an over-reacting of the sympathetic nervous system, involving a hypersensitivity of vascular baroreceptors in response to orthostasis [3–5]. As far as we are concerned, OHT has potential risk to induce cerebrovascular events and sustained arterial hypertension. According to previous publications, the relevant studies concentrating on OHT mostly focused on the elderly population [6]. Additionally, it is also involved in young and middle-aged people, but rarely reported in children. Concerning the incidence rate of OHT, there is no unified conclusion. In early 1992, Rutan et al. indicated that the incidence rate of OHT in healthy pilots was 4.2% [7]. In 2012, Wang Lili et al. reported that the incidence rate of OHT in elderly patients with hypertension was 9.0% [8]. In 2012, Chinese scholar Du Junbao proposed the diagnostic criteria of OHT in children, and analyzed their clinical features [9]. According to this diagnostic criteria, in 2013, Kang et al. analyzed the clinical information of children (n = 2089, age range 2.0–17.9) with syncope, headache, dizziness, chest tightness, and sigh. And the data illustrated that the total detection rate of OHT was 23.8%, males were higher than females (25.9% versus 21.6%, P < 0.05), 12-year-old group higher than < 12-year-old group (28.1% versus 20.5%, P < 0.01) [10]. Additionally, OHT is closely associated with the occurrence and development of persistent hypertension, cardiovascular events, diabetes, chronic kidney disease, asymptomatic cerebral infarction, and deep white matter ischemic lesions [11]. Therefore, it is particularly important to utilize appropriate blood pressure measurement methods to evaluate the changes of OHT. The 24-h semiautomatic ambulatory blood pressure monitoring can fully reflect the 24-h blood pressure changes and circadian rhythm of subjects, and it is an effective tool for objectively evaluating blood pressure changes. This technique can reduce the measuring error in traditional BP measurement methods, and avoid the influence of “white coat effect” [12]. According to the Consensus of Adolescent Children issued by the American Heart Association in 2014, the 24-h semiautomatic ambulatory blood pressure monitoring is effective in the diagnosis of white coat hypertension, asymptomatic hypertension, prehypertension, isolated diastolic hypertension, and nocturnal hypertension [13]. At present, there are few reports at home and abroad about the application of 24-h semiautomatic ambulatory blood pressure monitoring to evaluate the OHT type in children. In this case–control study, the 24-h semiautomatic ambulatory blood pressure monitoring was utilized to analyze OHT types in the Second Xiangya Hospital of Central South University, which provided reference for the diurnal blood pressure change of OHT in children.

## Methods

### OHT diagnostic criteria of the identified children

The normal blood pressure measured 10 min after supine in a quiet environment is considered to be basal blood pressure. And the OHT changes were measured 3 min after upright tilt test (HUTT, tilt angle 60). If the systolic blood pressure increased ≥ 20 mmHg, and (or) the diastolic blood pressure increased ≥ 10 mmHg (1 mmHg = 0.133 kPa), when compared with basal blood pressure, the patients could be diagnosed with OHT [9].

### Inclusion and exclusion criteria of OHT

(1) From October 2009 to September 2013, children at the syncope clinic of our hospital, or hospitalized children with unexplained syncope and premonitory syncope. (2) Patients were diagnosed with OHT via HUTT. (3) Physical examination, blood biochemical examination (fasting blood glucose, myocardial enzyme), conventional electrocardiogram, Holter electrocardiogram, echocardiography, electroencephalogram, cranial CT and MRI were carried out, in order to exclude heart, lung, brain, kidney and thoracic wall diseases.

### Study design

In the same period, healthy children in the Children’s Health Clinic of Xiangya Second Hospital of Central South University were selected into this research, and were matched with the OHT group (one-to-one based on age and sex). The identified patients were divided into two groups, and received basic HUTT (BHUT) and sublingual nitroglycerin tilt test (SNHUT), respectively. The electric tilting bed (ST-711) manufactured by Beijing Juchi Medical Technology Co., Ltd. and the tilting test monitoring software system (SHUT-100) provided by Beijing STADLEY Technology Inc. were utilized in this study. The specific inspection method refers to the guidelines for children’s syncope diagnosis issued by the Chinese Medical Association [14].

### Semiautomatic ambulatory blood pressure monitoring

During the semiautomatic ambulatory blood pressure monitoring examination, the subjects were home at 24 h. The ABPM 6100 monitor is provided by Welch Allyn in America, and the detailed information is as follows. (1) Select the same wide cuff and the same arm as semiautomatic ambulatory blood pressure monitoring. (2) Use a mercury sphygmomanometer to measure BP for 2 times, and then record BP value. (3) Initialize the BP monitoring and then enter the subject’s personal information. (4) The subjects were required to normatively wear BP monitoring, and manually measure blood pressure for 1 or 2 times. If the difference of systolic blood pressure between mercury sphygmomanometer and electronic sphygmomanometer less than 5 mmHg, the electronic sphygmomanometer could be considered to meet the requirements. Meanwhile, explain to the participants the precautions of wearing BP monitoring, and remind the required work and rest time. (5) After semiautomatic ambulatory blood pressure monitoring for 24 h, the blood pressure information of the subjects was input into the computer [15]. (6) Referring to the revised semiautomatic ambulatory blood pressure monitoring criteria for healthy children in 1997, the ambulatory blood pressure data can be achieved [16]. In this study, 6:00 to 22:00 was set as the daytime awakening time, and 22:00 to 6:00 in the next day was set as the nighttime sleep time. The blood pressure was monitored once every 30 min during the day and night, and the total monitoring time was not less than 23 h throughout the day. The BP judgment standard refers to the authoritative literature [17]. If the number of effective blood pressure less than 80%, it must be repeated every other day. In terms of matters needing attention, choose non-dominant arm to receive BP measuring. Secondly, determine cuff size based on the subject’s upper arm circumference. Thirdly, when the semiautomatic ambulatory blood pressure monitoring automatically measures BP, the upper arm of the cuff should be kept as static as possible. Fourthly, during semiautomatic ambulatory blood pressure monitoring, should avoid bathing, agitation, anxiety, strenuous exercise, and ban drinking and other diets that affect autonomic function. Fifthly, during BP monitoring, children’s daily routines and activities should be recorded, such as when they wake up, sleep, and take activities. At last, the subjects cannot stop BP monitoring at will [18]. Besides, the parameters of semiautomatic ambulatory blood pressure monitoring are as follows. (1) Average systolic BP (diastolic BP) = total value of systolic or diastolic BP in each time period/number of measuring times during each period. Calculate the mean systolic and diastolic BP at 24 h, in the daytime, and in the nighttime. (2) Diurnal variation of systolic BP = (mean systolic BP in the daytime − mean systolic BP in the nighttime)/mean systolic BP in the daytime × 100%. (3) Diurnal variation of diastolic BP = (mean diastolic BP in the daytime − mean diastolic BP in the nighttime)/mean diastolic BP in the daytime × 100%.

### Types of blood pressure

**Fig. 1:**
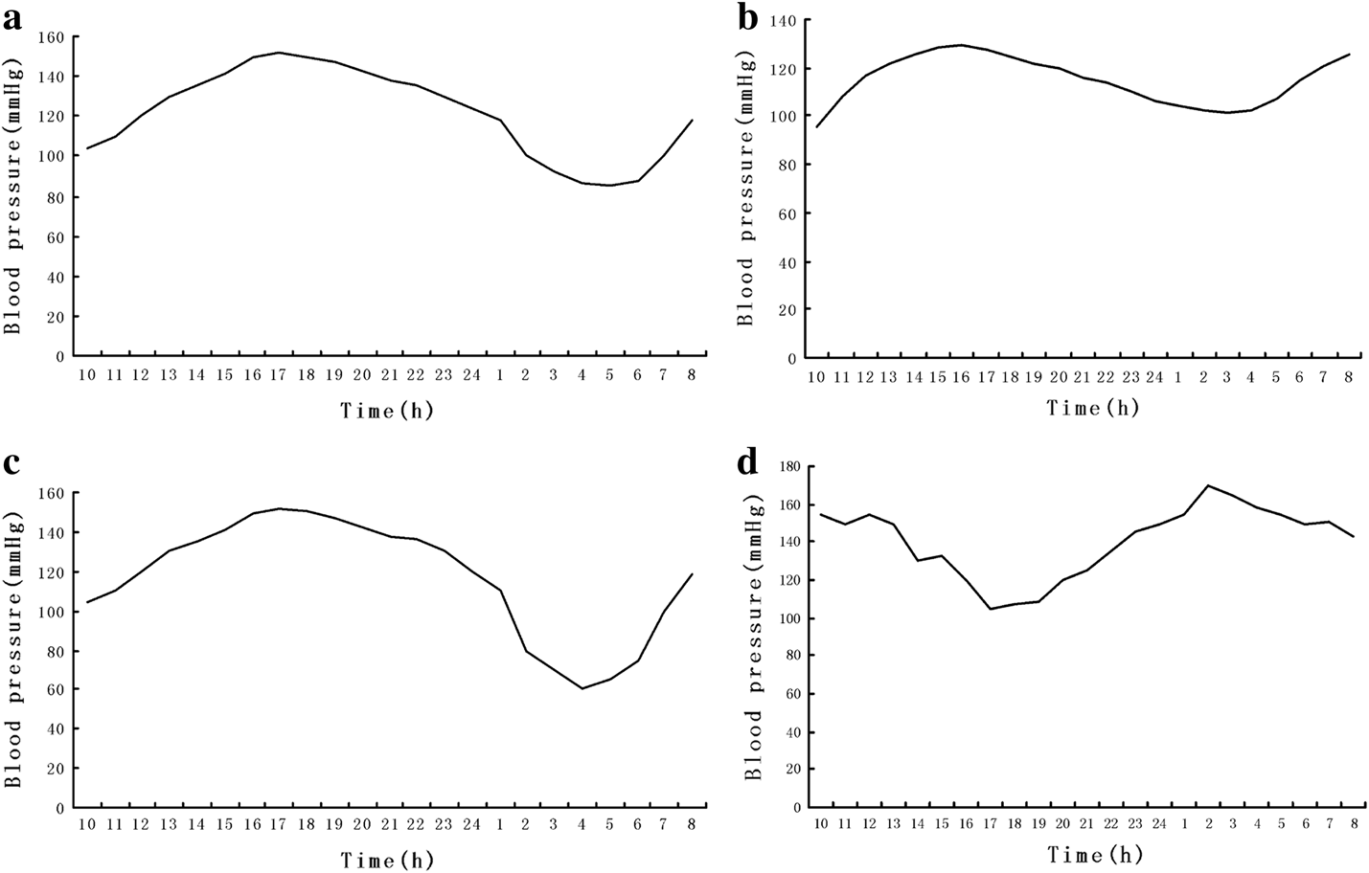
Diagram of blood pressure type. **a** Dipper blood pressure; **b** non-dipper blood pressure; **c** over spoon-shaped blood pressure; **d** inverse spoon-shaped blood pressure

When concerning the types of blood pressure, the thorough explanations are as follows. Under normal circumstances, the human body’s BP rises throughout the day and falls during the night, which can be described as “long handle spoon” curve [19]. (1) Dipper BP: Nocturnal BP drop > 10%, when compared with daytime BP, as shown in Fig. 1a. (2) Non-dipper BP: Nocturnal BP drop < 10%, when compared with daytime BP, as shown in Fig. 1b. (3) Over spoon-shaped BP: Nocturnal BP drop > 20%, when compared with daytime BP, as shown in Fig. 1c. (4) Inverse spoon-shaped BP: Nocturnal BP does not drop or increase, as shown in Fig. 1d.

### Statistical analysis

The statistical analysis was done by SPSS 21.0 Software. Quantitative variables were illustrated as mean ± SD, the comparisons between groups were carried out via independent sample t test. The qualitative variables as percentages, and the comparisons between groups were conducted with Chi square test. P < 0.05 was considered statistically significant.

## Results

### General characteristics of the identified patients

**Fig. 2:**
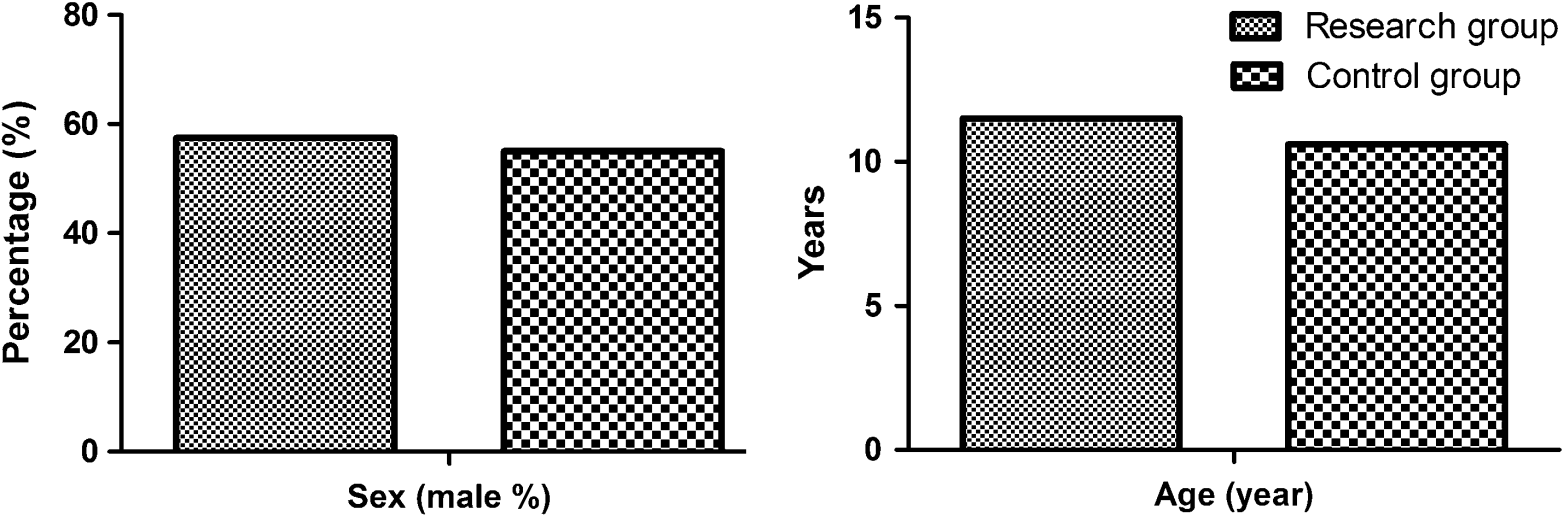
General characteristics of the identified patients

**Fig. 3:**
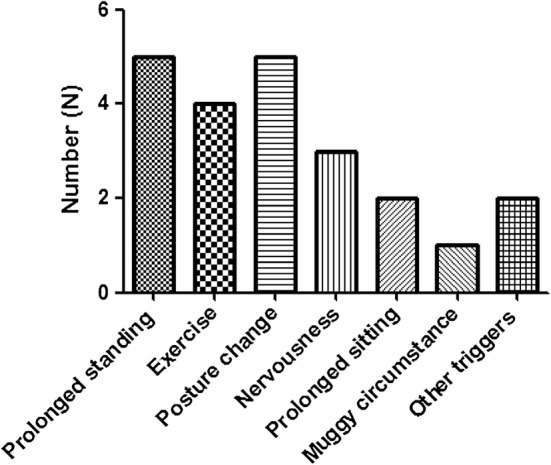
Common inducing factors that might resulted in OHT

40 cases in OHT group, 23 boys and 17 girls, age varied from 7 to 14 years old, mean age 11.5 ± 1.9 years old. 40 patients in the control group, 22 boys and 18 girls, age ranged from 4 to 14 years old, mean age 10.6 ± 2.4 years old. There was no significant differences in age and sex between the two groups (P = 0.081 and 0.822, respectively), as shown in Table 1 and Fig. 2. In addition, the primary clinical symptoms of the identified patients in OHT group included cardiovascular system, nervous system, and digestive system, as shown in Table 2. With respect to the common inducing factors that might resulted in OHT, the authors summarized them in this section, including prolonged standing, exercise, posture change, nervousness, prolonged sitting, muggy circumstance, other triggers, as shown in Fig. 3.

**Table 1:** Comparison of ABPM between OHT group and control group ($\bar{\chi }$ ± s, mmHg)

Group	24 h mean SBP	24 h mean DBP	Daytime mean SBP	Daytime mean DBP	Nocturnal mean SBP	Nocturnal mean DBP	SBP circadian difference	DBP circadian difference
OHT (n = 40)	107.2 ± 8.4	58.8 ± 6.1	11.2 ± 8.9	63.2 ± 7.0	101.8 ± 7.8	52.7 ± 5.7	8.4 ± 4.7	16.4 ± 7.1
Control (n = 40)	106.0 ± 8.6	58.2 ± 4.8	111.0 ± 9.2	63.2 ± 6.2	100.1 ± 8.4	52.2 ± 4.4	11.4 ± 3.1	16.9 ± 6.3
t	0.607	0.488	0.124	0.034	0.936	0.396	− 3.391	− 0.363
P	0.595	0.218	0.735	0.702	0.724	0.129	0.033	0.409

*BP* blood pressure, *SBP* systolic blood pressure, *DBP* diastolic blood pressure

**Table 2:** Comparison of heart rate variability between research group and control group

Indicator	Control group	Research group	t	P
SDNN (ms)	121.31 ± 32.54	154.23 ± 9.56	3.795	0.000
SDANN (ms)	122.96 ± 5.40	142.68 ± 3.21	4.127	0.002
rMSSD (ms)	76.08 ± 3.21	77.52 ± 3.62	1.238	0.301
pNN50 (%)	26.30 ± 4.52	27.51 ± 2.36	1.542	0.181
CV	0.12 ± 0.000	0.12 ± 0.000	0.310	0.823
TP	9532.21 ± 452.13	12,311.25 ± 411.23	3.421	0.001
ULF	4231.10 ± 369.21	6987.48 ± 398.41	4.259	0.000
VLF	2896.41 ± 231.41	3961.80 ± 357.12	2.361	0.010

### Parameter comparison of blood pressure between research group and control group

As shown in Table 3, the average systolic BP and diastolic BP in the OHT group at 24 h, in the daytime, and in the nighttime were slightly higher than those in the control group, but the difference was not statistically significant (P > 0.05). In addition, the difference of systolic BP between day and night in the control group was higher than that of the OHT group (P = 0.033), and the difference of diastolic BP between day and night in the control group was slightly higher than that of the OHT group (P = 0.409).

**Table 3:** Comparison of heart rate variability between boy and girl in research group

Indicator	Boy group	Girl group	t	P
Age (year)	12.01 ± 0.31	12.89 ± 0.28	1.891	0.089
SDNN (ms)	155.30 ± 3.21	142.58 ± 3.48	3.008	0.003
SDANN (ms)	148.21 ± 3.86	139.11 ± 4.50	0.956	0.451
rMSSD (ms)	79.55 ± 3.85	78.21 ± 3.69	0.781	0.487
pNN50 (%)	31.25 ± 1.26	29.91 ± 1.69	1.112	0.361
CV	0.11 ± 0.00	0.10 ± 0.00	1.460	0.189
TP	11,231.12 ± 445.21	1023.15 ± 397.13	3.921	0.002
ULF	6541.20 ± 451.23	4895.27 ± 401.23	2.361	0.002
VLF	3789.40 ± 230.78	2891.47 ± 309.87	3.561	0.001

### Comparison of blood pressure types between research group and control group

**Fig. 4:**
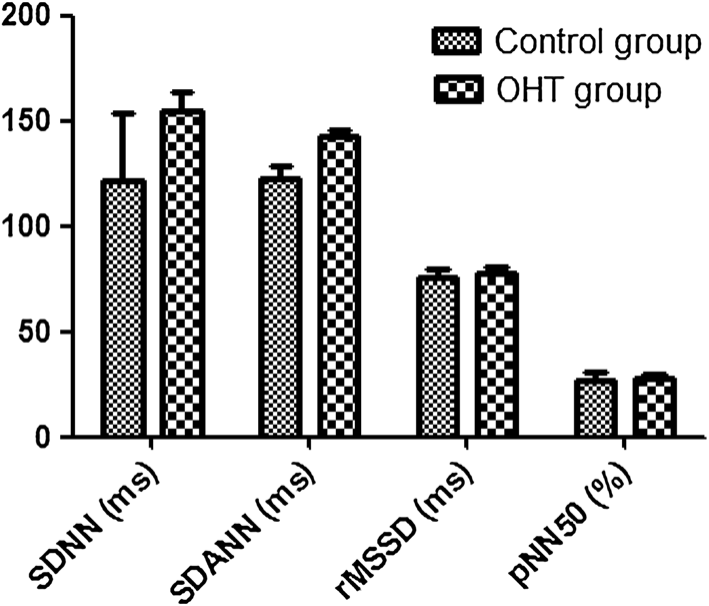
Comparison of heart rate variability between research group and control group

In the OHT group, non-dipper BP accounted for 72.5% (29/40 cases), and dipper BP accounted for 27.5% (11/40 cases). On the contrary, the non-dipper BP accounted for 45.0% (18/40 cases), and dipper BP accounted for 55.0% (22/40 cases). The BP difference between the two groups was statistically significant (χ^2^ = 6.600, P = 0.012). No over spoon-shaped BP and inverse spoon-shaped BP could be observed in both OHT and control groups, as shown in Table 4 and Fig. 4.

**Table 4:** Comparison of heart rate variability between research group and control group

Indicator	Control group	Research group	t	P
SDNN (ms)	121.31 ± 32.54	154.23 ± 9.56	3.795	0.000
SDANN (ms)	122.96 ± 5.40	142.68 ± 3.21	4.127	0.002
rMSSD (ms)	76.08 ± 3.21	77.52 ± 3.62	1.238	0.301
pNN50 (%)	26.30 ± 4.52	27.51 ± 2.36	1.542	0.181
CV	0.12 ± 0.000	0.12 ± 0.000	0.310	0.823
TP	9532.21 ± 452.13	12,311.25 ± 411.23	3.421	0.001
ULF	4231.10 ± 369.21	6987.48 ± 398.41	4.259	0.000
VLF	2896.41 ± 231.41	3961.80 ± 357.12	2.361	0.010

### Comparison of heart rate variability between research group and control group

**Fig. 5:**
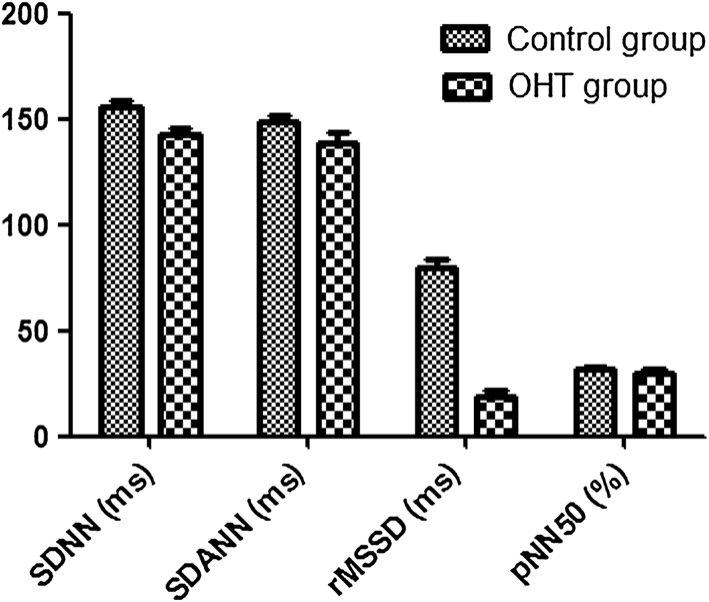
Comparison of heart rate variability between boy and girl in research group

AS shown in Table 2, the time domain SDNN and SDANN in the OHT group were higher than those in the control group (P < 0.01). In addition, the indicators including TP, ULF, VLF, and LF/HF were higher in the OHT group, when compared with control group (P < 0.01). In addition, in terms of subgroup analysis within the OHT group, the results were illustrated in Table 3. First of all, the age difference between boys and girls was not statistically significant (P > 0.05). When compared with girls, the time domain SDNN increased (P = 0.003), and the frequency index TP, ULF, and VLF increased in boy group (P < 0.05), as shown in Table 5 and Fig. 5.

**Table 5:** Comparison of heart rate variability between boy and girl in research group

Indicator	Boy group	Girl group	t	P
Age (year)	12.01 ± 0.31	12.89 ± 0.28	1.891	0.089
SDNN (ms)	155.30 ± 3.21	142.58 ± 3.48	3.008	0.003
SDANN (ms)	148.21 ± 3.86	139.11 ± 4.50	0.956	0.451
rMSSD (ms)	79.55 ± 3.85	78.21 ± 3.69	0.781	0.487
pNN50 (%)	31.25 ± 1.26	29.91 ± 1.69	1.112	0.361
CV	0.11 ± 0.00	0.10 ± 0.00	1.460	0.189
TP	11,231.12 ± 445.21	1023.15 ± 397.13	3.921	0.002
ULF	6541.20 ± 451.23	4895.27 ± 401.23	2.361	0.002
VLF	3789.40 ± 230.78	2891.47 ± 309.87	3.561	0.001

## Discussion

Perhaps on account of overcompensation for the gravitational challenge, the BP may rise when standing. In clinical practice, this phenomenon is called OHT, which is drawing increasing attention in recent years. It has been found to be associated with more prominent hypertensive target organ damage, such as silent cerebrovascular lesions. As mentioned above, no consensus has been reached on the definition of OHT, but OHT can be defined as a drop of SBP ≤ 20 mmHg and/or DBP ≤ 10 mmHg at orthostasis, according to the American Academy of Neurology. On the basis of our clinical experience, OHT is a common and disturbing problem and is especially prevalent among hospitalized patients. In addition, as a common clinical finding, it is closely associated with an increased risk of cardiovascular mortality, and is also an established marker for cardiovascular risk in patients with hypertension in the general population.

Arterial BP in the human body keeps changing along with blood flow, and there is a significant circadian rhythm in general. In terms of physiological basis, sympathetic nerves predominate during daytime in normal children, which enhances the concentration of catecholamines in plasma. The vagus nerve predominates during sleep at night, and the concentration of catecholamines in blood decreases. In further, when the body is in a supine position during sleep, redistribution of systemic blood flow occurs, and a large amount of blood concentrates in the lower limbs, which subsequently increase blood flow to the center, increased central venous pressure, increase afferent impulses in baroreceptors, and increase inhibitory stimulation of afferent vasomotor centers. Afterwards, the sympathetic excitatory reflex is weakened, the cardiac output decreases when the heart negatively inverts, and then the peripheral vascular resistance decreases and the whole body muscle relaxes, which subsequently results in lower BP levels during the night than during the day.

The circadian rhythm of blood pressure is one of the most important features of the human body. Maintaining a normal circadian rhythm of blood pressure is of great significance for protecting vital organs such as heart, liver and kidney, etc. For normal children, nocturnal BP is lower than daytime BP. Through the BP curve, BP is at its lowest point between 0–3 at early morning, starts to rise at 4–5 in the morning, and peaks between 6 and 8 in the morning. Afterwards, the BP is gradually steady, peaks again at 4–6 in the afternoon, and then slowly decreases. The BP throughout 24 h is presented as “long spoon-shaped curve” with double peaks and one valley. The semiautomatic ambulatory blood pressure monitoring can better reflect the BP fluctuation and circadian rhythm. The “over spoon-shaped” BP is often accompanied by OHT, and the “inverse spoon-shaped” BP is related to continuous sympathetic nerve stimulation, often accompanied by OHT [20]. Our team is dedicated to investigating the ambulatory BP changes in children with OHT and vasovagal syncope, and the corresponding results demonstrated that the BP type in children with OHT is mostly non-dipper BP and is associated with autonomic dysfunction [21]. In this study, the data illustrated that the most frequent BP type was non-dipper BP in children with OHT, which is consistent with the findings of Moriguchi et al. [22]. Wu et al. attempted to investigate the prevalence of hypertension in adults and its related factors (n = 1638), and they put forward the idea that OHT risk was closely related to changes of systolic BP during postural changes [23]. In this study, 80 cases were identified, and they received semiautomatic ambulatory blood pressure monitoring afterwards. The data demonstrated that the average BP in the OHT group was slightly higher than that in the control group, but the difference was not statistically significant. After comparisons between the two groups, it could be claimed that the pathogenetic mechanism of OHT, such as increased sympathetic activity and increased adrenaline boost sensitivity, could result in the increased BP in children with OHT, when compared with healthy children.

## Conclusion

In summary, the data in this research revealed that the BP type in children with OHT was mainly non-dipper BP, and the healthy children in the control group were mainly spoon-type BP. The difference between the two groups was statistically significant (P < 0.05). Additionally, the difference of systolic BP between day and night in the control group was greater than in the OHT group (P < 0.05). In brief, on account of autonomic dysfunction, the BP type in children with OHT was mostly non-dipper BP, and the circadian rhythm of BP disappeared.
